# Upfront surgery in patients with epithelial ovarian cancer and enlarged supradiaphragmatic lymph nodes associated with comparable to neoadjuvant chemotherapy

**DOI:** 10.1186/s12905-022-02082-5

**Published:** 2022-12-19

**Authors:** Omer Weitzner, Yael Yagur, Yfat Kadan, Ami Fishman, Rivka Zissin, Emilie Ben-Ezry, Limor Helpman, Mario E. Beiner

**Affiliations:** 1 Division of Gynecologic Oncology, Meir Medical Center, 59 Tchernichovsky St., Kfar Saba, Israel; 2 Clalit Health Services, Tel Aviv, Israel; 3 Sackler School of Medicine, Tel Aviv University, Tel Aviv, Israel; 4 Haemek Medical Center, Afula, Israel; 5 Rabin Medical Center, Petach Tikva, Israel; 6 Sheba Medical Center, Tel Hashomer, Israel

**Keywords:** Epithelial ovarian cancer stage III-IV, Enlarged supradiaphragmatic lymph nodes, Chest recurrences, Upfront cytoreduction, Neoadjuvant chemotherapy

## Abstract

**Background:**

There is little data regarding the optimal approach to advanced epithelial ovarian cancer (EOC) with isolated extra-peritoneal disease in the cardiophrenic lymph nodes. This study assessed whether the prognosis and surgical outcomes are affected by the treatment approach among these patients.

**Material and methods:**

This retrospective cohort study included patients with advanced EOC, who were treated 2012–2020. Computed tomography scans were reviewed for disease extent and the presence of enlarged supradiaphragmatic nodes (SDLN). Demographic, clinical and oncologic data were recorded. Characteristics and outcomes of patients with and without enlarged SDLN were evaluated, and outcomes of patients with enlarged SDLN who underwent upfront surgery and neoadjuvant chemotherapy were compared.

**Results:**

Among 71 women, 47 (66%) had enlarged supradiaphragmatic lymph nodes. Groups had similar baseline characteristics. Among 47 women who had enlarged SDLN. There was no significant difference in progression free survival among patients who had upfront cytoreduction compared to those who received neoadjuvant chemotherapy. Only one asymptomatic chest recurrence was observed.

**Conclusion:**

Patients with enlarged SDLN have comparable outcomes with either upfront surgery or neoadjuvant chemotherapy. Moreover, the frequency of chest recurrences in patients presenting with enlarged SDLN is exceedingly low.

## Introduction

Epithelial ovarian cancer (EOC) is the fifth leading cause of cancer-related deaths in women in the United States and carries a high case-fatality rate [1]. Patients are commonly diagnosed at an advanced stage and are treated with surgery and chemotherapy [2]. Evaluation usually includes imaging of the chest, abdomen, and pelvis, to establish the extent of disease and the feasibility of optimal surgical resection [3]. Supradiaphragmatic lymph nodes (SDLN), also called paracardiac or cardiophrenic nodes, are positioned just above the diaphragm. In advanced EOC, where the peritoneal tumor burden is often large, these lymph nodes are a frequent site of metastases [4, 5]. Suspicious nodes are usually defined as larger than 5–10 mm in the short axis diameter and are commonly identified on axial imaging, such as computed tomography (CT) scans.[4, 6, 7] The appropriate treatment approach for patients with enlarged SDLN has not been established [4, 6, 8–10] although some recent evidence suggests that they can be removed surgically [11, 12] and vascular procedures can be safely performed with proper pre-operative planning [13].

Considering the paucity of clinical data on the effect of surgery on patients with advanced EOC with isolated extra-peritoneal disease in the cardiophrenic nodes, this single institution, retrospective, cohort study was undertaken to explore the effect of different treatment approaches on the outcomes of patients with advanced EOC and enlarged SDLN.

## Materials and methods

This retrospective cohort study compared patients with advanced EOC with or without isolated extra-peritoneal disease in the cardiophrenic nodes who underwent surgery at a single center, 2012 through 2020. Surgical procedures included hysterectomy, bilateral salpingo-oophorectomy, omentectomy, peritonectomies, diaphragmatic stripping and bowel resections, as required and at the surgeon’s discretion. SDLN were not resected.

Patients were identified from a prospectively-collected institutional database and included all stage III patients and stage IV patients with extraperitoneal disease limited to the supradiaphragmatic nodes, with histologically proven EOC and availability of adequate preoperative imaging. Patients with rapidly progressing disease not allowing initiation of treatment were excluded. CT scans were retrospectively reviewed by the same radiologist for disease extent and enlarged SDLN, which were defined as larger than 5 mm in the short axis [14]. Clinical and biochemical data were extracted from patient charts and electronic medical records and included patient age, BRCA status, CA-125 level at diagnosis, tumor histology, timing of surgery and residual tumor.

The tumor burden was defined in both groups of patients based on the Eisenkop score, according to the surgical findings. Eisenkop et al. [15] developed a score specific for ovarian cancer that is based on the spread of peritoneal and retroperitoneal disease.

The primary outcome evaluated was progression free survival (PFS), defined as the interval between the last course of first-line chemotherapy administered and radiologically documented recurrence. PFS was compared for patients categorized according to presence or absence of enlarged SDLN and for patients with enlarged SDLN categorized by treatment approach: upfront surgery or neoadjuvant chemotherapy followed by interval debulking surgery.

### Ethical approval

This study was performed in accordance with the principles of the Declaration of Helsinki. All methods were carried out according to relevant guidelines and regulations. All experimental protocols were approved by the Meir Medical Center Human Investigation Committee, number MMC-0183-17. Due to the retrospective nature of the study, Meir Medical Center Human Investigation Committee waived the need for informed consent.

### Statistical analysis

Student *t*-test was used to compare continuous variables, and chi-square and Fisher's exact test for categorical variables, each as appropriate. Analyses were two-sided, and results were considered significant when the *P*-value was ≤ 0.05. Data are presented as numbers and percentages for categorical variables and as medians for continuous variables. Time-to-event outcomes, such as PFS, were plotted using the Kaplan–Meier method and compared using the log-rank test. All statistical analyses were performed using the Statistical Package for the Social Sciences for Windows, Version 24.0 (IBM Corp., Armonk, NY).

## Results

The study cohort included 71 women who met the inclusion criteria. Among these patients, 47 (66%) had enlarged SDLN at presentation, and 24 (34%) did not. Among those with enlarged SDLN, 20 (43%) had bilateral nodes, 22 had nodes on the right side (47%) and 5 (10%) on the left side only. The baseline characteristics of the study patients grouped by SD lymph node status are presented in Table 1. There were no significant differences between the two groups with respect to patient age, timing of surgical debulking or neoadjuvant treatment, or residual peritoneal tumor at the end of debulking surgery. Supradiaphragmatic nodes were not surgically removed at the time of surgery. Those with enlarged SDLN had higher CA125 levels at diagnosis (mean 1,797 ± 357.5 U/ml vs. 708 ± 98.6 U/ml, *P* = 0.04, OR 2.47, 95% CI 1.14–5.34), although the tumor burden was similar in both groups based on Eisenkop scores according to the surgical findings [15].

**Table 1:** Baseline characteristics

Parameter	Enlarged supradiaphragmatic lymph nodes		*P*-value
	Present N = 47	Absent N = 24	
Age, years (mean ± SD)	62.4 ± 8.7	65.7 ± 10.1	0.158
*BRCA status, n* (%)			
Unknown	12 (25.6%)	6 (25%)	0.714
BRCA1 positive	8 (17%)	6 (25%)	
BRCA1 negative	27 (57.4%)	12 (50%)	
CA-125 at diagnosis, U/ml (mean ± SD)	1797.3 ± 357.5	708.2 ± 98.6	0.048
*Surgery, n* (%)			
Upfront	22 (46.8%)	9 (37.5%)	0.454
Interval	25 (53.2%)	15 (62.5%)	
*Histology, n* (%)			
Serous	43 (91.6%)	23 (95.8%)	0.773
Endometrioid	1 (2.1%)	0 (0%)	
Mucinous	1 (2.1%)	0 (0%)	
Carcinosarcoma	1 (2.1%)	1 (4.2%)	
Squamous cell	1 (2.1%)	0 (0%)	
Tumor burden* *(average Eisenkop score)*	4.4 ± 2.0	4.5 ± 2.1	0.854

*At completion of cytoreductive surgery

**Fig. 1:**
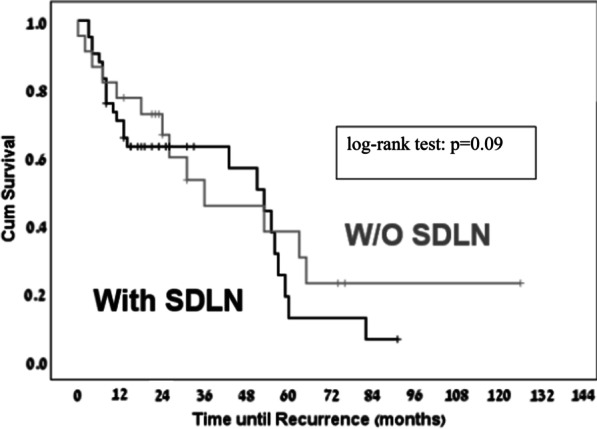
Time to recurrence based on presence or absence of enlarged supradiaphragmatic lymph nodes (SDLN)

There was no statistically significant difference in PFS between patients grouped by the presence or absence of enlarged SDLN, despite a trend toward better PFS among patients without enlarged SDLN (median 19.6 vs. 12.6 months, *P* = 0.09, OR 1.44, 95% CI 0.94–2.34; Fig. 1).

Table 2 presents the outcome according to treatment modality (primary surgery vs. neoadjuvant chemotherapy and interval debulking) and the presence of enlarged SDLN. Optimal debulking (> 10 mm) was achieved in most patients in both groups.

**Table 2:** Outcomes according to treatment modality and presence of enlarged supradiaphragmatic lymph nodes

	Neoadjuvant treatment		*P*-value	Upfront surgery		*P*-value
	Enlarged SDLN N = 19	No enlarged SDLN N = 9		Enlarged SDLN N = 28	No enlarged SDLN N = 15	
Progression free survival (median, months)	9.7	16.5	0.09	15.1	21.5	0.11
% Optimal debulking* (< 10 mm)	73.6% (14/19)	100% (9/9)	0.79	85.7% (24/28)	100% (15/15)	0.81
% No macroscopic disease	57%(11/19)	89%(8/9)	0.09	78%(22/28)	93%(14/15)	0.06

*Of intraperitoneal disease

**Fig. 2:**
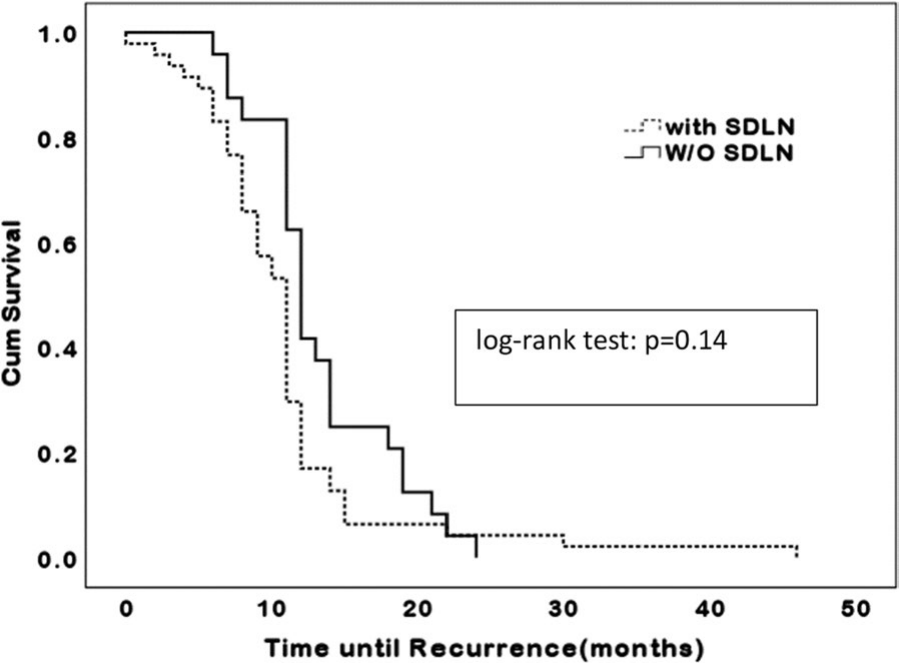
Progression free survival among patients with no macroscopic residual tumor in the presence or absence of enlarged supradiaphragmatic lymph nodes (SDLN)

**Fig. 3:**
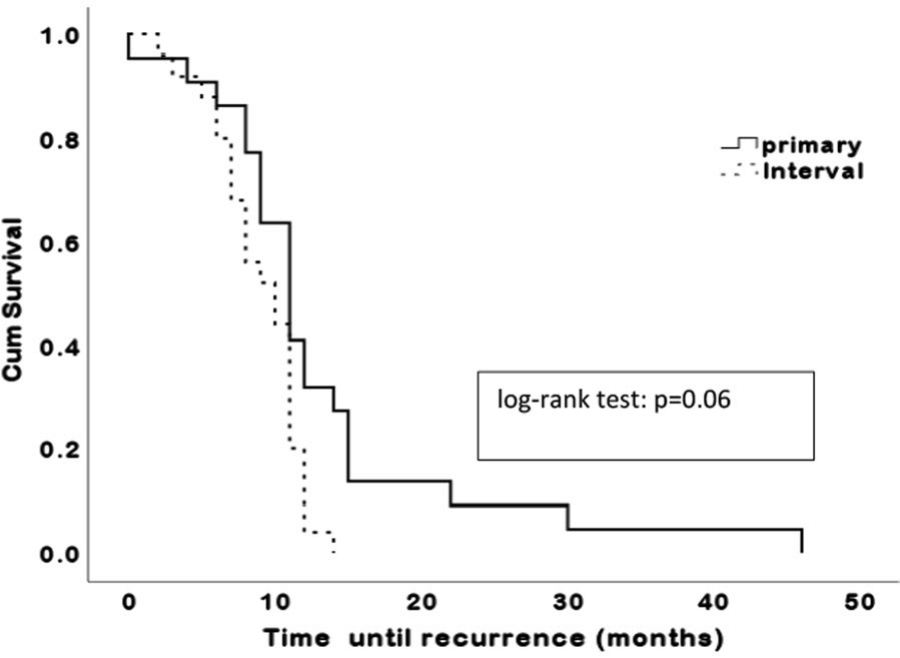
Progression free survival among patients with enlarged supradiaphragmatic lymph nodes (SDLN) having primary or interval surgery

A trend toward longer PFS was seen in patients without enlarged SDLN in both upfront surgery and neoadjuvant chemotherapy groups, but the difference was not statistically significant (median 21.5 vs. 15.1 months after upfront surgery, log-rank test: *P* = 0.11, OR 1.66, 95% CI 0.74–1.94 and median 16.5 vs. 9.7 months after neoadjuvant chemotherapy, log-rank test: *P* = 0.09, OR 2.17, 95% CI 0.90–2.84; Table 2 and Figs. 2, 3).

A total of 34 patients experienced disease recurrence, 24 (51%) in the group with enlarged SDLN and 10 (41.6%) in the group without SDLN. The most common sites for recurrence were intraperitoneal (29 patients, 85.2%), para-aortic lymph nodes (2, 5.8%), brain (2, 5.8%) and chest (1, 2.9%). The patient with chest recurrence was in the group with enlarged SDLN. Here again, a trend toward longer PFS was seen in patients without enlarged SDLN in both upfront surgery and neoadjuvant chemotherapy groups, but the difference was not statistically significant (median 21.5 vs. 15.1 months after upfront surgery, log-rank test: *P* = 0.11, OR 1.66, 95% CI 0.74–1.94 and median 16.5 vs. 9.7 months after neoadjuvant chemotherapy, log-rank test: *P* = 0.09, OR 2.17, 95% CI 0.90–2.84). Among patients with enlarged SDLN, there was no statistically significant difference in PFS for those having upfront cytoreduction compared to those receiving neoadjuvant chemotherapy (log- rank test median 17.1 vs. 12.2 months, *P* = 0.06, OR 1.86, 95% CI 0.64–1.88 (Fig. 3), although in both groups the SDLN were not removed during surgery.

## Discussion

This study explored the treatment outcomes of patients with stage III-IV EOC, with and without enlarged SDLN, at a single institution. Despite a trend toward less favorable outcomes, PFS was not statistically different for women with SDLN at presentation compared to women with disease limited to the peritoneum. Chest recurrences among these women were rare. Only one patient had a supra-diaphragmatic recurrence concurrent with an intraperitoneal recurrence. Moreover, PFS among women with enlarged SDLN did not appear to be associated with surgical approach and was comparable for patients who underwent upfront cytoreduction and those who received neoadjuvant chemotherapy for high tumor burden in the initial evaluation followed by interval cytoreduction.

The relatively high rate of SDLN could be because CT scans were reviewed retrospectively by a radiologist for disease extent and enlarged SDLN.

Several studies have investigated the association between enlarged SDLN and prognosis in patients with advanced EOC. Kolev et al. evaluated 212 patients with stage III-IV ovarian cancer who underwent primary cytoreductive surgery. Among them, 92 (43%) with enlarged SDLN, demonstrated a trend toward poorer overall survival but without a statistically significant difference (45 vs. 50 months, *P* = 0.09). When analyzing overall survival among patients who underwent optimal cytoreduction, the same trend was observed (50 vs. 55 months, *P* = 0.09) [7].

Raban et al. compared 31 patients with stage IIIC EOC and enlarged SDLN to 41 controls. Patients with enlarged nodes were more likely to be treated with neoadjuvant chemotherapy (71% vs. 26.8%, *P* < 0.001). Disease free survival in patients with enlarged SDLN was significantly shorter (9 vs. 24 months, *P* = 0.0097); although, the results were not stratified by primary treatment modality [9].

Prader et al. compared 133 EOC patients who had radiologically normal cardiophrenic lymph nodes (CPLN) to 217 patients who had enlarged/suspicious CPLN. In patients with postoperative residual tumor, enlarged CPLN had no impact on survival. However, CPLN were associated with a lower complete resection rate: in patients with no gross residual disease, CPLN metastases were associated with shorter PFS (5-year PFS 13% vs. 41%, log-rank *P* < 0.001) and overall survival (OS) (5-year OS 30% vs. 69%, log-rank test, *P* = 0.009) [16].

Other studies have also reported conflicting data regarding the impact of CPLN on prognosis in EOC [17, 18] and the clinical relevance of enlarged CPLN seen on staging CT of patients with EOC [19].

The role of surgical cytoreduction in stage IV EOC has been supported by several observational studies [11, 12, 20, 21]. There are several reports on the feasibility of resecting enlarged SDLN during cytoreductive surgery [22, 23]. The procedure seems to be safe when performed in the proper setting and does not delay chemotherapy [10–12]. Although resection of bulky lymph nodes in gynecologic oncology is a challenging procedure, there is evidence that in carefully selected patients with gynecologic cancer with bulky lymph nodes, laparoscopic lymph node debulking could be considered a valid option to avoid potential severe vascular intraoperative complications [24]. However, some evidence refutes the role of supra-diaphragmatic cytoreduction in this disease [10]. It is known that the site of relapse is closely related to the primary location, regardless of the type of initial treatment [25]. Our data suggest that most disease recurrences are abdominal, even in the absence of supra-diaphragmatic cytoreductive efforts. Further studies are needed to determine the impact of this intervention on outcomes. The best treatment option for ovarian cancer recurrence is still subjective, can vary among centers and depends on personal experience [26].

Bevacizumab was used for maintenance treatment for patients in both groups with residual disease after surgery or stage IV [13].

The major strengths of the current study are the inclusion of a homogeneous group of ovarian cancer patients who underwent surgery at a single center from 2012 to 2020. Their CT scans were retrospectively reviewed by the same experienced radiologist for disease extent and for the presence of enlarged SDLN. This is also one of few studies evaluating the association of enlarged SDLN with treatment outcomes in an unselected cohort of patients with stage III-IV EOC. To our knowledge, it is the first study to compare outcomes according to treatment modality (upfront cytoreductive surgery vs. neoadjuvant chemotherapy followed by interval surgery) and by the extent of residual disease at cytoreductive surgery.

Limitations are inherent to the retrospective nature of this study, including incomplete documentation of patient follow-up in the medical record and missing information, including lack of overall survival data. Another important limitation was the size of the patient population, which may have affected our ability to have statistically significant observations. The study was underpowered to detect a significant difference in the primary outcome, and a sample size of 437 patients would be required to detect a 6-month difference in PFS between groups. Interestingly, for the comparison of PFS in patients with enlarged SDLN by treatment approach, not even a trend for a negative association with upfront surgery was observed.

Our data did not demonstrate a statistically significant difference in PFS among patients with advanced EOC presenting with or without SDLN. Moreover, in patients with enlarged SDLN, we found no association between treatment approach and outcomes: primary cytoreduction appeared to be comparable to neoadjuvant chemotherapy followed by interval cytoreduction. However, the analysis was underpowered and definitive conclusions cannot be drawn from these observations. Further research powered to detect a clinically significant association is needed to determine the most appropriate primary treatment for these women, including the value of removing enlarged SDLN as part of cytoreductive surgery.

## Conclusions

PFS among women with enlarged SDLN did not appear to be associated with surgical approach and was comparable for patients undergoing upfront cytoreduction and those receiving neoadjuvant chemotherapy followed by interval cytoreduction. Moreover, despite a trend toward less favorable outcomes, PFS was not found to be statistically different for women with SDLN at presentation compared to women with disease limited to the peritoneum, and chest recurrences among these women were rare.

## Data Availability

The datasets used and/or analyzed during the current study are available from the corresponding author on reasonable request.

## Abbreviations

EOC: Epithelial ovarian cancer
SDLN: Supradiaphragmatic nodes
CT: Computed tomography
PFS: Progression free survival
CPLN: Cardiophrenic lymph nodes
